# Nostochopcerol, a new antibacterial monoacylglycerol from the edible cyanobacterium *Nostochopsis lobatus*

**DOI:** 10.3762/bjoc.19.13

**Published:** 2023-02-09

**Authors:** Naoya Oku, Saki Hayashi, Yuji Yamaguchi, Hiroyuki Takenaka, Yasuhiro Igarashi

**Affiliations:** 1 Biotechnology Research Center and Department of Biotechnology, Toyama Prefectural University, 5180 Kurokawa, Imizu, Toyama 939-0398, Japan; 2 MAC Gifu Research Institute, MicroAlgae Corporation, 4-15 Akebono, Gifu 500-8148, Japan

**Keywords:** antibacterial, cyanobacterium, edible, monoacylglycerol, *Nostochopsis lobatus*

## Abstract

A new antibacterial 3-monoacyl-*sn*-glycerol, nostochopcerol (**1**), was isolated from a cultured algal mass of the edible cyanobacterium *Nostochopsis lobatus* MAC0804NAN. The structure of compound **1** was established by the analysis of NMR and MS data while its chirality was established by comparison of optical rotation values with synthetically prepared authentics. Compound **1** inhibited the growth of *Bacillus subtilis* and *Staphylococcus aureus* at MIC of 50 μg/mL and 100 μg/mL, respectively.

## Introduction

Cyanobacteria are widely accepted as a prolific source of unique bioactive metabolites [1]. Some cyanobacterial species are consumed as food, nutritional supplements, or folk medicines in many parts of the world [2–3], and have offered attractive opportunities for drug discovery. Results from the limited number of attempts include an antifungal lipopeptide nostofungicidine [4] and an antioxidant nostocionone [5] from *Nostoc commune*, an unusual antibacterial *n*−1 fatty acid from *N. verrucosum* [2], and the sacrolides, antimicrobial oxylipin macrolactones from *Aphanothece sacrum* [6–7].

*Nostochopsis lobatus* is a freshwater species distributed in every climate zone but polar regions [8]. It grows on riverbed rocks or cobbles in shallow streams and forms spherical to irregularly lobed, hollow, gelatinous colonies, with sizes reaching up to 5.5 cm in diameter [9]. Although cosmopolitic, its occurrence is dominated in tropical regions, thus food consumption of this alga is only reported from India [10] and Thailand [11]. In India, local tribes utilize it as a dietary supplement [10]. In northern Thailand, this alga occurs in dry season from November to April and is called *Lon*, *Kai Hin* (stone egg), or *Dok Hin* (stone flower) [11]. It is consumed as an ingredient of salad and as a folk medicine to treat pain from stomach ulcers or fever [9]. In fact, an ethanolic extract of the air-dried alga was found to inhibit the development of gastric ulcers, suppress ethyl phenylpropiolate-induced edema on ear, and decrease writhing response induced by intraperitoneal injection of acetic acid in rodent models [11], thus supporting the ethnophamacological testimonies. Moreover, radical scavenging activity [11–12], hyaluronidase inhibitory activity [13], and tyrosinase inhibitory activity [14] were detected by in vitro testings, which further raised the expectation of its richness as the source of bioactive metabolites. However, at present, only a single drug discovery attempt has been made on this alga [13], which prompted further chemical study.

**Figure 1:**
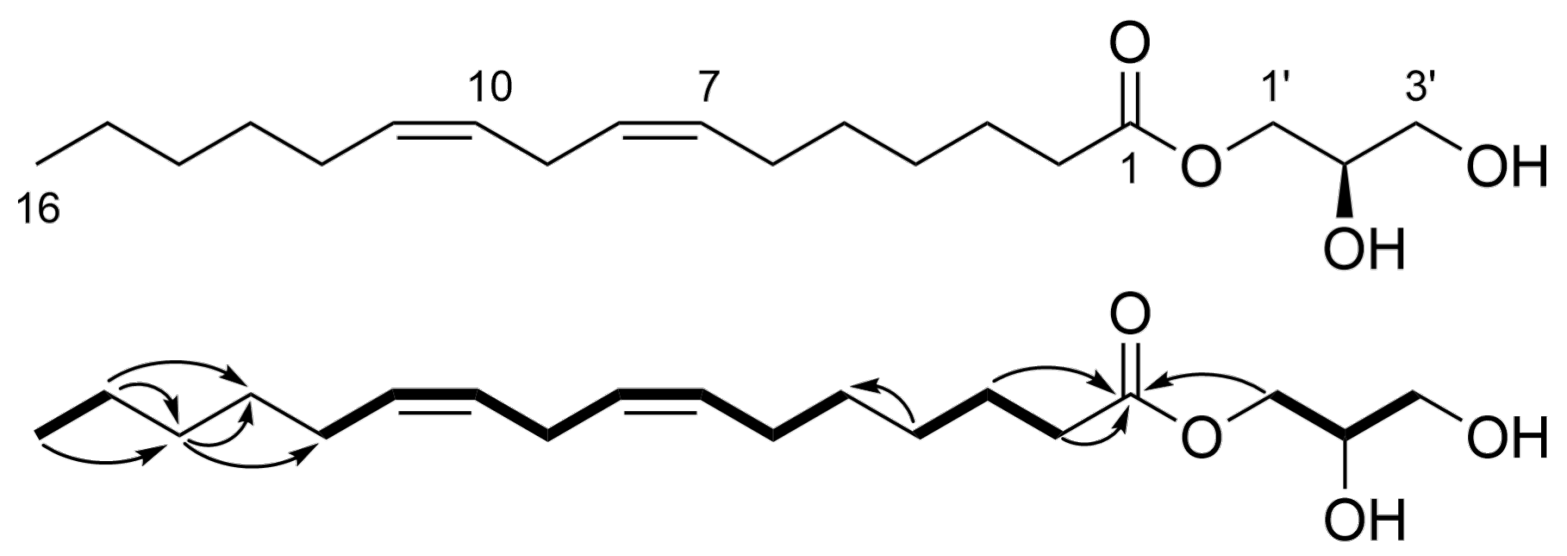
Structure of nostochopcerol (**1**) and selected COSY (bold lines) and HMBC (arrows) correlations.

We evaluated the antimicrobial activity of the ethanolic extract of this alga and found that a mid-polar fraction inhibited the growth of two Gram-positive bacteria, *Bacillus subtilis* and *Staphylococcus aureus*. Activity-guided fractionation led to the discovery of a new monoacylglycerol, nostochopcerol (**1**, Figure 1). Part of this study have been described in a patent [15].

## Results and Discussion

A water-thawed algal mass of strain MAC0804NAN (374.6 g) was repeatedly extracted with EtOH. The combined extract was partitioned between 60% aqueous MeOH and CH_2_Cl_2_, and the latter lipophilic layer was further partitioned between 90% aqueous MeOH and *n*-hexane. The resulting three layers were tested against four Gram-positive bacteria, five Gram-negative bacteria, six fungi, and two yeasts, which detected antibacterial activity against two Gram-positive bacteria, *Bacillus subtilis* and *Staphylococcus aureus*, from the 90% aqueous MeOH layer. The responsible constituent, though prone to diffuse during chromatography, was purified with the guidance of antibacterial activity on ODS and Sephadex LH-20 and by reversed-phase HPLC on ODS and styrene-divinylbenzene copolymer to yield 0.7 mg of compound **1** from 113.3 mg of the solvent partition fraction. The reason for the low yield of compound **1** was eventually understood after it was determined to be a monoacylglycerol, which has a surface-active property and should have deteriorated the separation capacity of the chromatographic resins.

The molecular formula of compound **1** was established to be C_19_H_34_O_4_ based on a sodium adduct pseudomolecular ion at *m*/*z* 349.2348 [M + Na]^+^ observed by high-resolution ESITOFMS (calcd for C_19_H_34_NaO_4_^+^, 349.2349). Three degrees of unsaturation, calculated from the molecular formula, were accounted for by a carboxyl group (δ_C_ 175.3) and two double bonds (δ_C_ 130.9, 130.6, 129.1, and 128.9) observed in the ^13^C NMR spectrum (Table 1), revealing that compound **1** has a linear structure. The ^1^H NMR spectrum contained resonances typical of an unsaturated fatty acid, such as non-conjugated olefins with four-proton integration (δ_H_ ca. 5.34–5.32, 4H), a bisallylic methylene (δ_H_ 2.77, brt, *J* = 6.5 Hz, H_2_9), a methylene adjacent to a carboxyl group (δ_H_ 2.34, brt, *J* = 7.5 Hz, H_2_2), two allylic methylenes (δ_H_ 2.07, H_2_6 and 2.05, H_2_12), and an aliphatic methyl group (δ_H_ 0.89, t, *J* = 6.9 Hz, H_3_16). Along with these resonances, several oxygenated (δ_H_ ca. 4.13–3.54) and aliphatic signals (δ_H_ 1.62 and ca. 1.39–1.30) were observed, implying that compound **1** is a derivative of a fatty acid. Indeed, all oxygenated protons constituted a spin system (CH_2_1'–CH2'–CH_2_3') in the COSY spectrum (Figure 1), and considering the lack of any terminal group besides CH_3_16, monoacylglycerol was the only possible structure for compound **1**. This assignment was eventually proven after interpretation of the whole set of 1D and 2D NMR data. A carboxy carbon, four sp^2^ methines, one oxymethine, two oxymethylenes, ten aliphatic methylenes, and a methyl group were collected from the analysis of ^13^C NMR and HSQC spectra and these structural pieces were assembled into four spin systems by the COSY correlations: an ethyl group (C16–C15), a C_8_ internal hydrocarbon chain with two degrees of unsaturation (C12–C11=C10–C9–C8=C7–C6–C5), three consecutive methylenes (C4–C3–C2) with a carboxy-termination, and a glyceryl moiety (Figure 1). The *Z*-geometry was deduced for both double bonds (Δ^7^ and Δ^10^) from shielded chemical shift values of the allylic carbons (C6: 27.9 ppm and C12: 28.0 ppm) [16]. The first two COSY fragments were connected via the intervention of two methylene groups (CH_2_13 and CH_2_14) by five HMBC correlations H14/C12, H14/C13, H15/C13, H15/C14, and H16/C14, while the second and third fragments were directly connected by a correlation from H4 to C5. The (7*Z*,10*Z*)-hexadecadienoyl unit thus constructed settled C_16_H_27_O_2_ of the molecular formula, leaving C_3_H_7_O_2_ for the glyceryl group. Finally, interconnection of the acyl and glyceryl units via an ester linkage was verified by three HMBC correlations from the terminal protons (H1, H2, and H1') of both units to the carboxy carbon (C1), leaving two protons to occupy C2' and C3' diol. Thus, compound **1** was determined to be a new monoacylglycerol and named nostochopcerol after the source organism.

**Table 1:** ^1^H (500 MHz) and ^13^C (125 MHz) NMR data for nostochopcerol (**1**) in CD_3_OH (δ in ppm).

Position	δ_C_	δ_H_, mult. (*J* in Hz), integr.	HMBC (^1^H to ^13^C)

1	175.3		2, 3
2	34.8	2.34, t (7.5), 2H	3
3	25.8	1.62, qui (7.4), 2H	2
4	29.7	1.36, ovl, 2H	2, 3, 5, 6
5	30.29	1.39, ovl, 2H	
6	27.9	2.07, m, 2H	7
7	129.1	5.338, m, 1H	9
8	130.6	5.335, m, 1H	6, 9
9	26.4	2.77, brt (6.5), 2H	8, 10
10	128.9	5.32, m, 1H	9, 12
11	130.9	5.34, m, 1H	9, 12
12	28.0	2.05, m 2H	11
13	30.34	1.303, ovl, 2H	
14	32.5	1.296, ovl, 2H	12, 13, 16
15	23.5	1.310, ovl, 2H	14, 16
16	14.3	0.89, t (6.9), 3H	15
1'	66.4	4.05, dd (6.3, 11.3), 1H	1, 2', 3'
		4.13, dd (4.4, 11.3), 1H	1, 2', 3'
2'	71.2	3.80, m, 1H	
3'	64.1	3.54, brs, 2H	

**Scheme 1:**
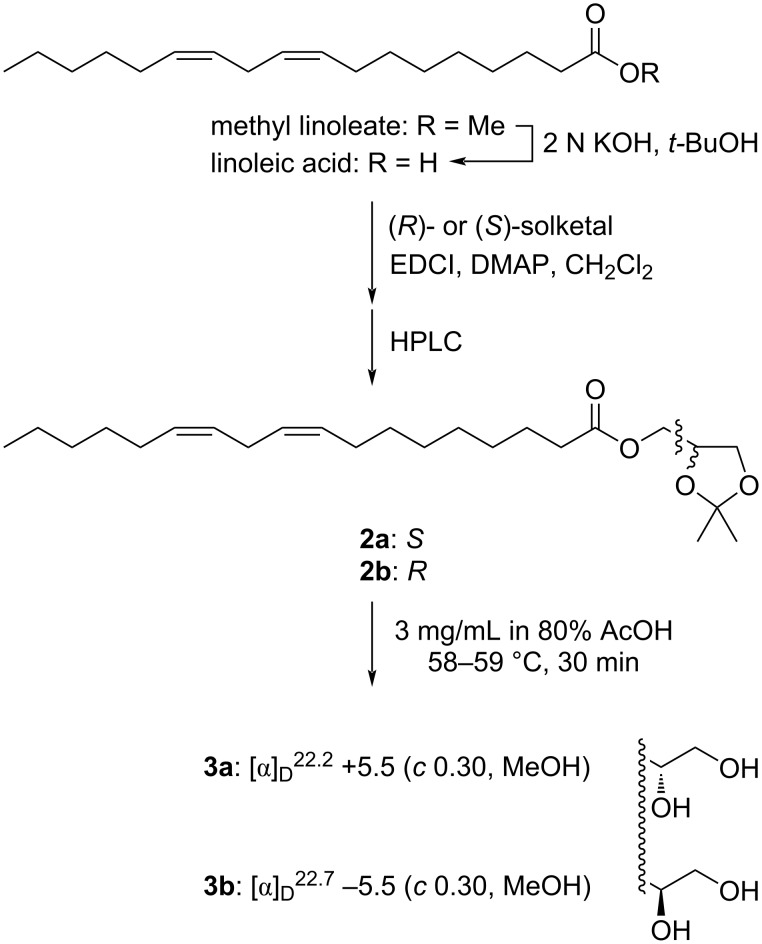
Synthesis of 1-linoleoyl-*sn*-glycerol (**3a**) and 3-linoleoyl-*sn*-glycerol (**3b**).

The absolute configuration of the sole chiral center at C2' in the glyceryl group was addressed by comparing the optical rotation value of compound **1** with those of synthetically prepared authentic chiral monoacylglycerols. Because (7*Z*,10*Z*)-hexadecadienoic acid was not commercially available, methyl linoleate, having the same degree of unsaturation with a longer chain length by two carbons, was used as a source of the acyl chain. Linoleic acid, obtained by saponification of methyl linoleate, was condensed either with (*R*)- or (*S*)-solketal (isopropylidene glycerol) by Steglich esterification. The resulting ester **2a** or **2b** was purified by reversed-phase HPLC and deprotected by a short treatment with 80% aqueous acetic acid at 58–59 °C to give 1-linoleoyl-*sn*-glycerol (**3a**) or 3-linoleoyl-*sn*-glycerol (**3b**), respectively (Scheme 1). Similarly, to our experience during the isolation of compound **1**, swapping the order of purification and deprotection severely decreased the yields (data not shown). The *sn*-1-acyl isomer **3a** exhibited a positive rotation ([α]_D_^22.3^ +5.5 (*c* 0.30, MeOH)) while the *sn*-3-acyl isomer **3b** gave a negative rotation ([α]_D_^22.5^ −5.5 (*c* 0.30, MeOH)), suggesting that compound **1** is acylated at *sn*-3 as judged by its negative value ([α]_D_^22.4^ −5.9 (*c* 0.01, MeOH)).

Compound **1** is the first non-glycosylated glycerolipid isolated from cyanobacteria [17–20]. Natural 3-acylated-*sn*-glycerols were also reported from the fungus *Sclerotinia fructicola* [21] and a brown alga *Ishige sinicola* [22]. The (7*Z*,10*Z*)-hexadecadienoyl group has been found in galactoglycerolipids from *Chlorella* [23–25], kale (*Brassica oleracea*) [26], *Daphnia* [27], and meadow buttercup (*Ranunculus acris*, family *Ranunculaceae*) [28], and as a sucrose ester from rough horsetail (*Equisetum hiemale*, phylum *Pteridophyta*) [29].

Monoacylglycerols are non-ionic surfactants derivable by hydrolysis of fat, and exhibit antibacterial [30], antifungal [30], antiviral [31], and antiprotozoal [32] activities. Due to these useful properties, they have found a wide range of industrial applications as emulsifiers, antifoamers, preservatives, antistatic agents, polymer lubricants, and mold-releasing agents for the production of foods, cosmetics, ointments, paints, and plastics [33]. Compound **1** exhibited antibacterial activity, evaluated by a microculture method, with MIC 50 μg/mL against *B. subtilis* ATCC6633 and 100 μg/mL against *S. aureus* FDA209P JC-1 (Table 2). Congener **3b**, having a two-carbon-longer alkyl chain, was equally potent against *S*. *aureus* but was less active against *B*. *subtilis* than compound **1**. Interestingly, *sn*-3 linoleate **3b** was more potent than its antipode **3a**.

**Table 2:** Antimicrobial activity of nostochopcerol (**1**) and synthetic analogs.

Compound	*Bacillus subtilis*	*Staphylococcus aureus*

**1**	50	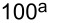
**3a**	>200	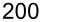
**3b**	200	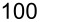
kanamycin sulfate^b^	5	0.63

^a^Minimum inhibitory concentration (μg/mL) at which the growth was completely inhibited. ^b^Positive control.

## Experimental

### General methods

Cosmosil 75C18-PREP (Nacalai Tesque Inc., 75 µm) was used for ODS flash chromatography. NMR spectra were obtained on a Bruker AVANCE II 500 spectrometer using residual solvent peaks at δ_H_/δ_C_ 3.30/49.0 ppm in CD_3_OH and 7.27/77.0 ppm in CDCl_3_ as chemical shift reference signals. HR-ESITOFMS analysis was conducted on a Bruker micrOTOF mass spectrometer. Optical rotation and UV spectra were recorded on a JASCO P-1030 polarimeter and a Shimadzu UV-1800 spectrophotometer, respectively.

### Biological material

*N. lobatus* MAC0804NAN was cultured as described in [13].

### Extraction and isolation

A water-thawed specimen (374.6 g) was homogenized with an equal amount of Celite in EtOH (400 mL). The resulting slurry was paper-filtered to afford an ethanolic extract and an algal cake, and the latter was extracted three more times. The combined extract was concentrated in vacuo and the resulting suspension was diluted with MeOH to adjust its concentration to 60% (v/v). This was extracted with CH_2_Cl_2_ for three times, and the CH_2_Cl_2_-soluble layer was partitioned between aqueous 90% MeOH and *n*-hexane. The most active aqueous MeOH layer (113.3 mg) was subjected to ODS flash chromatography with a stepwise elution by MeCN/50 mM NaClO_4_ 30:70, 45:55, 60:40, 75:25, 90:10, and chloroform/MeOH/H_2_O 6:4:1 to give six fractions. Antibacterial activity against *S. aureus* FDA209P JC-1 and *B. subtilis* ATCC6633 was detected with the second and fourth fractions. The latter was gel-filtered on Sephadex LH-20 (MeCN/50 mM NaClO_4_ 75:25) to see the separation of activity at the top two and slow-eluting fractions. The top fraction was purified by repeated HPLC first on an ODS column (Cosmosil AR-II 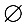 1 × 25 cm) and second on a styrene-divinylbenzene polymer column (Hamilton PRP-1 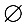 1 × 25 cm) both eluted with MeCN/50 mM NaClO_4_ 75:25 to yield compound **1** (0.7 mg).

Nostochopcerol (**1**): [α]_D_^22.4^ −5.9 (*c* 0.01, MeOH); UV (MeOH) λ_max_, nm (log ε): 200 (1.7); HRMS–ESIMS (*m/z*): [M + Na]^+^ calcd for C_19_H_34_NaO_4_**^+^**, 349.2349; found, 349.2348; IR (ATR) ν_max_: 3350, 2921, 2852, 1601, 1457, 1195, 1103, 1015, 875, 696 cm^−1^.

### Paper disk-agar diffusion method

According to a procedure described in [6], the antimicrobial potency of chromatographic fractions was evaluated by a paper disk-agar diffusion method. Fractions at each purification stage were diluted to the same concentration with MeOH, and 10 μL aliquots were impregnated into 6 mm-diameter paper disks, which were left standing until completely dried. A loop of the test organism, suspended in a small amount of water, was mixed with liquefied agar medium precooled to nearly body temperature, and the inoculated medium was quickly poured into a sterile plastic dish. The composition of the medium is 0.5% yeast extract, 1.0% tryptone, 1.0% NaCl, 0.5% glucose, and 1.5% agar. After the agar solidified, the drug-impregnated disks were placed on the medium, and the test cultures were incubated at 32 °C for a day or two until the diameters of inhibitory haloes turned measurable.

### Microculture antimicrobial testing

To each well of a sterile 96-well microtiter plate was dispensed 100 μL of tryptic soy broth. Additionally, 98 μL of the same medium and 2 μL of the solutions of test compounds in MeOH or a reference antibiotic, kanamycin monosulfate, in H_2_O, were added to the wells at the top row. To make two-fold serial dilutions along the column, 100 μL aliquots from the wells of the top row were taken and added to the well in the second row and mixed gently with the pre-dispensed medium by pipetting. In the same manner, 100 μL aliquots were transferred from the second row to the third row. This operation was repeated until the transfer of diluted drug solutions reached the bottom row. The excess 100 μL in the bottom row was discarded to equalize the volume of the medium in the wells. The test strains, *S*. *aureus* FDA209P JC-1 and *B*. *subtilis* ATCC6633, were recovered on tryptic soy agar, and a loopful of bacterial masses was transferred to tryptic soy broth in a 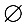 16 mm tube. The tubes were shake-cultured for several hours at 37 °C at 306 rpm until the turbidity measured by the absorbance at 600 nm (ABS_600_) exceeded 0.1. The liquid culture was diluted to adjust the turbidity to ABS_600_ 0.09–0.1 (0.5 McFarland), which corresponds to a cell density of 1.5 × 10^8^ cfu/mL. This was further diluted by 75 times to prepare a cell suspension of 2.0 × 10^6^ cfu/mL, of which 100 μL were dispensed to the wells to give microcultures with the final cell density of 1.0 × 10^6^ cfu/mL. The plates were incubated at 37 °C for 48 h and the concentration at which the growth of microbes was completely inhibited was defined as the minimum inhibitory concentration (MIC).

## Supporting Information

Supporting information features procedures for synthesis of chiral α-linoleoyl glycerols, physicochemical properties of synthetic compounds, HRESITOF mass spectrometric analysis of nostochopcerol (**1**), copies of NMR spectra for **1**, 3-linoleoyl-1,2-*O*-isopropylidene-*sn*-glycerol (**2b**), and 1-linoleoyl-*sn*-glycerol (**3a**).

File 1 Experimental details, characterization data and copies of spectra.
