# Supplementary material for: Nostochopcerol, a new antibacterial monoacylglycerol from the edible cyanobacterium Nostochopsis lobatus
Source: Beilstein J Org Chem. 2023 Feb 9;19:133–8. doi: 10.3762/bjoc.19.13 (PMC9924362; doi:10.3762/bjoc.19.13)
Supplement: File 1 — Experimental details, characterization data and copies of spectra. [file Beilstein_J_Org_Chem-19-133-s001.pdf]

BEILSTEIN JOURNAL OF ORGANIC CHEMISTRY

Supporting Information

for

Nostochopcerol, a new antibacterial monoacylglycerol from the edible cyanobacterium *Nostochopsis lobatus*

Naoya Oku, Saki Hayashi, Yuji Yamaguchi, Hiroyuki Takenaka and Yasuhiro Igarashi

Beilstein J. Org. Chem. **2023,** *19,* 133–138. doi:10.3762/bjoc.19.13

Experimental details, characterization data and copies of spectra

License and Terms: This is a supporting information file under the terms of the Creative Commons Attribution License (https://creativecommons.org/licenses/by/4.0). Please note that the reuse, redistribution and reproduction in particular requires that the author(s) and source are credited and that individual graphics may be subject to special legal provisions.
The license is subject to the *Beilstein Journal of Organic Chemistry* terms and conditions: (https://www.beilstein-journals.org/bjoc/terms)

Table of contents

Experimental procedure..........S2

Preparation of linoleic acid from methyl linoleate

Chiral α-linoleyl-α',β-*O*-isopropylidene glycerols **2a** and **2b**

Chiral α-linoleoylglycerols **3a** and **3b**

^{1}H (500 MHz) and ^{13}C (125 MHz) NMR data for compounds **2b** and **3a** in $CDCl_3$

MS and NMR spectra

nostochopcerol (**1**)

Positive ion HRESITOFMS spectrum S5

^{1}H NMR spectrum (CD_3OH, 500 MHz) S6

^{13}C NMR spectrum (CH_3OH, 125 MHz) S7

COSY spectrum (CH_3OH, 500 MHz) S8

HSQC spectrum (CH_3OH, 500 MHz) S9

HMBC spectrum (CH_3OH, 500 MHz) S10

3-linoleoyl-1,2-*O*-isopropylidene-*sn*-glycerol (**2b**)

^{1}H NMR spectrum ($CDCl_3$, 500 MHz) S11

^{13}C NMR spectrum ($CDCl_3$, 125 MHz) S12

COSY45 spectrum ($CDCl_3$, 500 MHz) S13

HSQC spectrum ($CDCl_3$, 500 MHz) S14

HMBC spectrum ($CDCl_3$, 500 MHz) S15

1-linoleoyl-*sn*-glycerol (**3a**)

^{1}H NMR spectrum ($CDCl_3$, 500 MHz) S16

^{13}C NMR spectrum ($CDCl_3$, 125 MHz) S17

COSY45 spectrum ($CDCl_3$, 500 MHz) S18

HSQC spectrum ($CDCl_3$, 500 MHz) S19

HMBC spectrum ($CDCl_3$, 500 MHz) S20

Preparation of linoleic acid from methyl linoleate

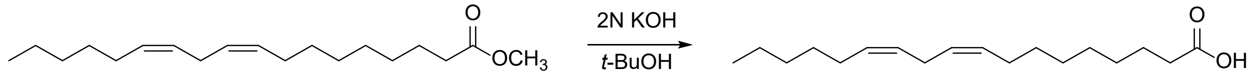

Methyl linoleate (1.00 g), purchased from Tokyo Chemical Industry Co., Ltd. (product code S0325), was dissolved in a 1:1 mixture of *t*-BuOH (5 mL) and 2 N KOH (3 mL) and the solution was stirred for 3 h at an ambient temperature. After removing *t*-BuOH under reduced pressure, the resulting residue was partitioned between EtOAc (20 mL) and 2 N HCl (20 mL). The EtOAc layer was successively washed with water and brine and then slowly passed through anhydrous Na_2SO_4 to give linoleic acid (1.04 g), which was used for the next step without further purification.

Chiral α-linoleyl-α',β-*O*-isopropylidene glycerols 2a and 2b

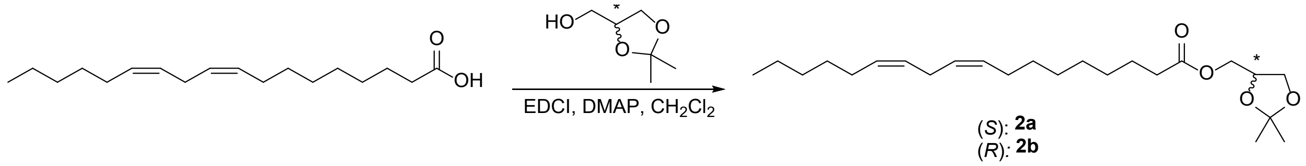

To the solution of linoleic acid (112.8 mg, 0.402 mmol for **2a**; 122.2 mg, 0.436 mmol for **2b**) in CH_2Cl_2 (3 mL) was added (*R*)- or (*S*)-solketal (2,2-dimethyl-1,3-dioxolane-4-methanol, Tokyo Chemical Industry, product codes D1705 and D1691, respectively: 59 mg, 0.45 mmol; 1.1 equiv), 1-ethyl-3-(3-dimethylaminopropyl)carbodiimide hydrochloride (86.9 mg, 0.45 mmol: 1.1 equiv), and one crystal of *N*,*N*-dimethyl-4-aminopyridine (DMAP, ≈ 5 mg), and the reaction mixture was stirred for 2.5 h at ambient temperature. After removing the solvent under reduced pressure, the resulting concentrate was passed through a short column of silica gel to remove DMAP and 1-(3-(dimethylamino)propyl)-3-ethylurea. The eluate was concentrated and purified by ODS-HPLC (solvent: 95%MeCN) to give 1-linoleoyl-2,3-*O*-isopropylidene-*sn*-glycerol (**2a**, 90.6 mg, 57.5%) and 3-linoleoyl-1,2-*O*-isopropylidene-*sn*-glycerol (**2b**, 125.0 mg, 72.7%), respectively [34].

1-Linoleoyl-2,3-*O*-isopropylidene-*sn*-glycerol (**2a**): $[\alpha]^{23.7}_D$ 0.69 (*c* 1.0, MeOH); HRESIMS: *m/z* 417.2969 $[M + Na]^+$ (calcd for $C_{24}H_{42}NaO_4$, 417.2975). IR (ATR) ν_{max} 3010, 2987, 2929, 2856, 1743, 1458, 1381, 1372, 1254 sh, 1215, 1161, 1087, 1059, 976, 844, 726 cm^{-1}. ^1H and ^{13}C NMR data are essentially the same as **2b**.

3-Linoleoyl-1,2-*O*-isopropylidene-*sn*-glycerol (**2b**): $[\alpha]^{23.7}_D$ −0.64 (*c* 1.0, MeOH); HRESIMS: *m/z* 417.2961 $[M + Na]^+$ (calcd for $C_{24}H_{42}NaO_4$, 417.2975). IR (ATR) ν_{max} 3010, 2987, 2929, 2857, 1743, 1458, 1381, 1372, 1254 sh, 1215, 1161, 1085, 1058, 977, 844, 727 cm^{-1}; ^1H and ^{13}C NMR, see Table S1.

Chiral α-linoleoylglycerols 3a and 3b

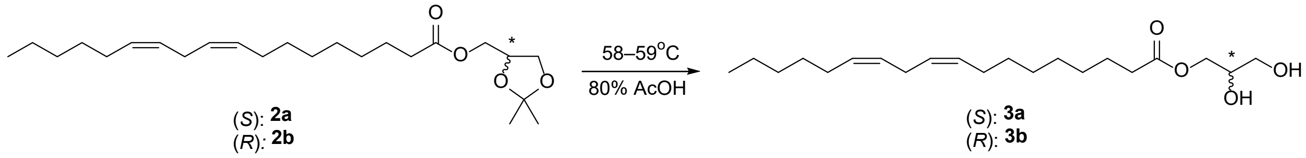

Compound **2a** (5.9 mg, 15.0 μmol) or **2b** (6.2 mg, 15.7 μmol) was dissolved in 80% aqueous acetic acid (2 mL) and the solution was stirred occasionally in a sealed 6-mL screw-capped vial at 58–59 °C for 30 min. After removing the solvents by a stream of N_2 gas, the resulting concentrate was purified by silica gel-HPLC (Cosmosil SL-II Ø 1 × 25 cm) eluted with *n*-hexane/EtOAc 1:1 (4 mL/min) monitored at 210 nm to give 1-linoleoyl-*sn*-glycerol (**3a**, 4.3 mg, 81.1%) or 3-

linoleoyl-*sn*-glycerol (**3b**, 5.1 mg, 91.6%), respectively [1-4].

1-Linoleoyl-*sn*-glycerol (**3a**): $[\alpha]^{22.2}_{D}$ +5.5 (*c* 0.30, MeOH); HRESIMS: *m/z* 377.2652 $[M + Na]^+$ (calcd for $C_{21}H_{38}NaO_4$, 377.2662). IR (ATR) ν_{max} 3404, 3010, 2927, 2856, 1740, 1458, 1378, 1243, 1177, 1118, 1052, 726 cm^{-1}; ^1H and ^{13}C NMR, see Table S1.

3-Linoleoyl-*sn*-glycerol (**3b**): $[\alpha]^{22.7}_{D}$ −5.5 (*c* 0.30, MeOH); HRESIMS: *m/z* 377.2642 $[M + Na]^+$ (calcd for $C_{21}H_{38}NaO_4$, 417.2662). IR (ATR) ν_{max} 3407, 3010, 2926, 2856, 1739, 1458, 1379, 1242, 1177, 1120, 1053, 723 cm^{-1}. ^1H and ^{13}C NMR data are essentially the same as for **3a**.

References

1. Zhang, G.-L.; Xing, Q.-Y.; Zhang, M.-Z. *Phytochemistry*, **1997,** *45,* 1213–1215.

2. Kim, C.; Ha, H.; Kim, J. S.; Kim, Y. T.; Kwon, S.-C.; Park, W.-W. *Arch. Pharm. Res.* **2003,** *26,* 34–39.

3. Fraser, B. H.; Perlmutter, P.; Wijesundera, C. *J. Am. Oil Chem. Soc.* **2007,** *84,* 11–21.

4. Degenhardt, A. G.; Hofmann, T.; *J. Agric. Food Chem.* **2010,** *58,* 12906–12915.

Table S1: ^{1}H (500 MHz) and ^{13}C (125 MHz) NMR data for compounds **2b** and **3a** in $CDCl_3$.

	**2b**		**3a**	
Position	δ_C	δ_H[a]	δ_C	δ_H[a]
1	173.6		173.4	
2	34.1	2.34, t (7.6), 2H	34.1	2.35, t (7.6), 2H
3	24.9	1.62, brqui (7.3), 2H	24.9	1.63, brqui (7.2), 2H
4	29.081[b]	1.30, ovl	29.12	1.3068, ovl
5	29.087[b]	1.30, ovl	29.06	1.3076, ovl
6	29.1	1.30, ovl	29.06	1.3076, ovl
7	29.3[c]	1.32, ovl, 2H	29.3[i]	1.31, ovl, 2H
8	27.19[d]	2.05, ovl, 2H	27.18[j]	2.05, ovl, 2H
9	130.2[e]	5.38[f], m, 1H	130.2[k]	5.38[l], m, 1H
10	128.1[g]	5.323[h], m, 1H	128.1[m]	5.348[n], m, 1H
11	25.6	2.77, brt (6.6), 2H	25.6	2.77, brt (6.6), 2H
12	127.9[g]	5.316[h], m, 1H	127.9[m]	5.32[n], m, 1H
13	130.0[e]	5.36[f], ovl, 1H	130.0[k]	5.355[l], ovl, 1H
14	27.18[d]	2.05, ovl, 2H	27.15[j]	2.05, ovl, 2H
15	29.6[c]	1.33, ovl, 2H	29.6[i]	1.34, ovl, 2H
16	31.5	1.29, ovl, 2H	31.5	1.29, ovl, 2H
17	22.6	1.30, ovl, 2H	22.5	1.302, ovl, 2H
18	14.1	0.89, t (7.0), 3H	14.0	0.89, t (6.9), 3H
sn-1	66.4	3.74, dd (6.2, 8.4), 1H	63.3	3.60, dd (5.8, 11.4), 1H
		4.07, dd (6.4, 8.6), 1H		3.70, dd (4.0, 11.5), 1H
sn-2	73.7	4.31, m, 1H	70.3	3.93, m, 1H
sn-3	64.5	4.09, dd (6.0, 11.5), 1H	65.2	4.15, dd (6.2, 11.7), 1H
		4.16, dd (4.7, 11.5), 1H		4.21, dd (4.7, 11.7), 1H
acetonide-O*C*O	109.8			
acetonide-CH_3-1	26.7	1.43, s, 3H		
acetonide-CH_3-2	25.4	1.37, s, 3H		

[a]Chemical shift in ppm, multiplicity (*J* in Hz), integral. [b-u]Interchangeable.

Mass Spectrum Molecular Formula Report

Analysis Info

Analysis Name	D:\Data\igarashi lab\Hayashi Saki\LC-MS\42A-22-2 HR\42A-22-2 pos-2_1_01_4295.d	Acquisition Date	2/2/2016 2:55:26 PM
Method	lcms_esi_pos_wide.m	Operator	Bruker Customer
Sample Name	42A-22-2 pos-2	Instrument / Ser#	micrOTOF 210
Comment			

Acquisition Parameter

Source Type	ESI	Ion Polarity	Positive	Set Nebulizer	1.2 Bar
Focus	Not active			Set Dry Heater	200 °C
Scan Begin	50 m/z	Set Capillary	4500 V	Set Dry Gas	10.0 l/min
Scan End	3000 m/z	Set End Plate Offset	-500 V	Set Divert Valve	Source

Generate Molecular Formula Parameter

Formula, min.	C18H32O3Na				
Formula, max.	C20H36O5Na				
Measured m/z	349.235	Tolerance	4 mDa	Charge	1
Check Valence	no	Minimum	0	Maximum	0
Nirogen Rule	no	Electron Configuration	both		
Filter H/C Ratio	no	Minimum	0	Maximum	3
Estimate Carbon	yes				

Sum Formula	Sigma	m/z	Err [ppm]	Mean Err [ppm]	Err [mDa]	rdb	N Rule	e⁻
C 19 H 34 Na 1 O 4	0.009	349.2349	0.24	0.36	0.08	2.50	ok	even

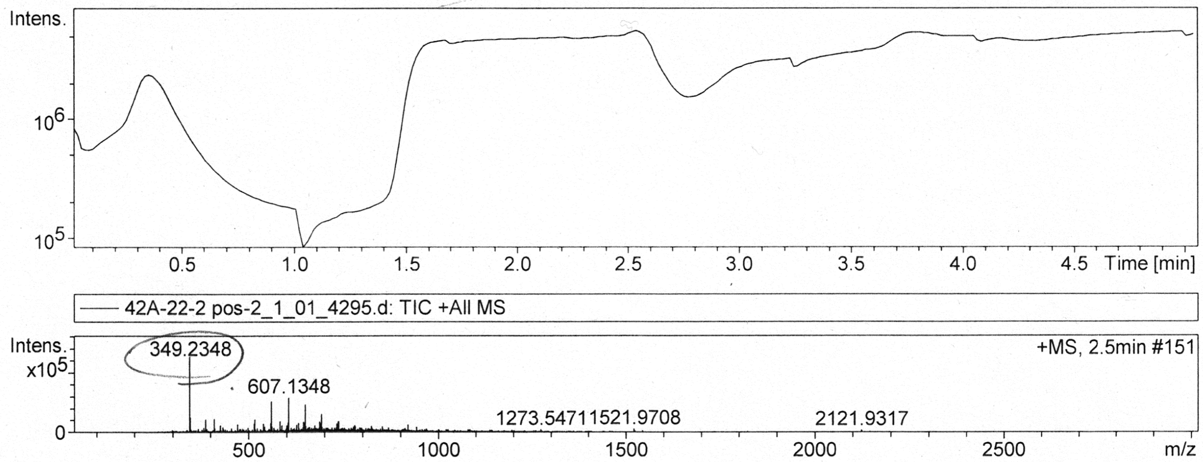

Positive ion HRESITOFMS spectrum of nostochopcerol (**1**)

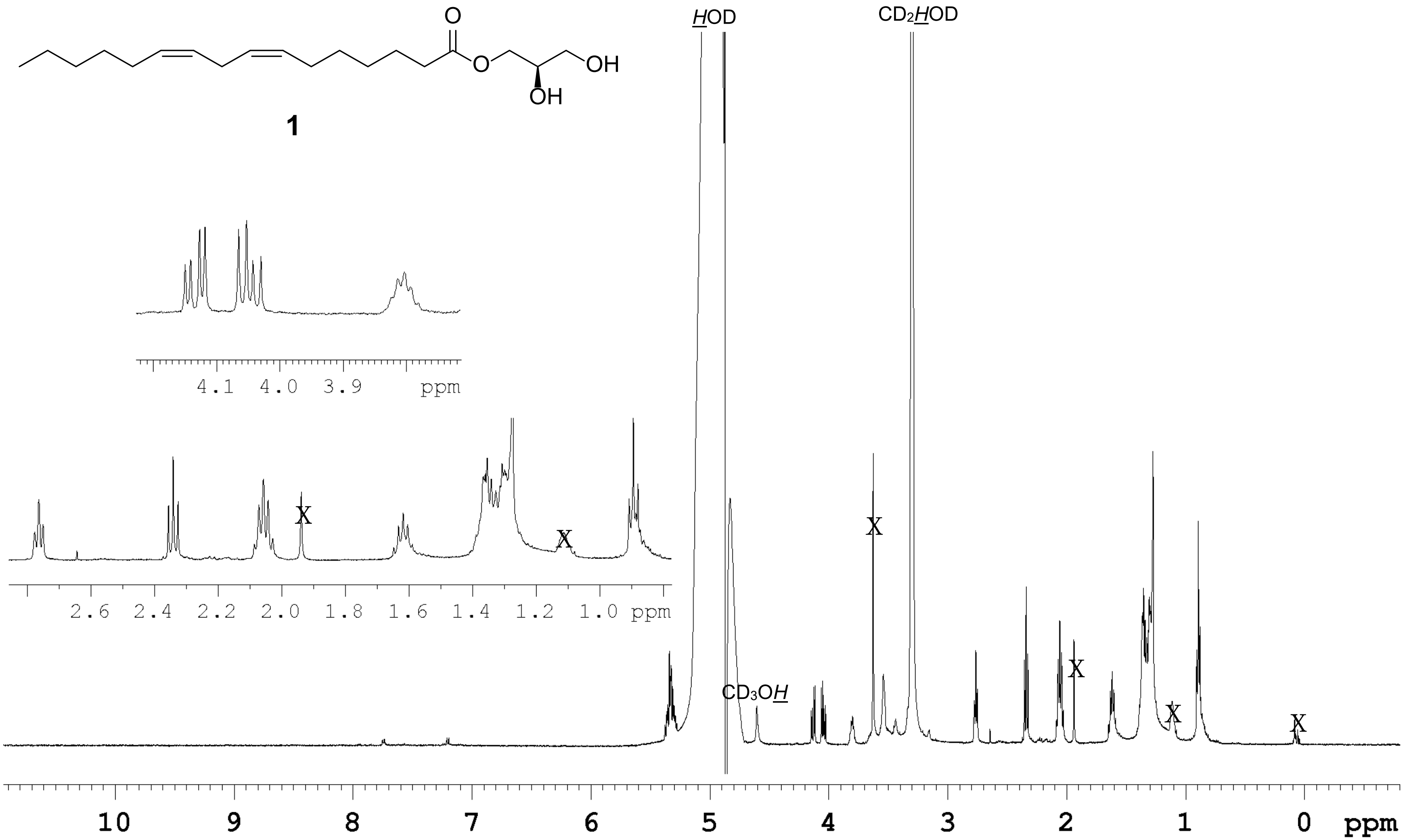

^{1}H NMR spectrum of nostochopcerol (**1**) (500 MHz, CD_3OH)

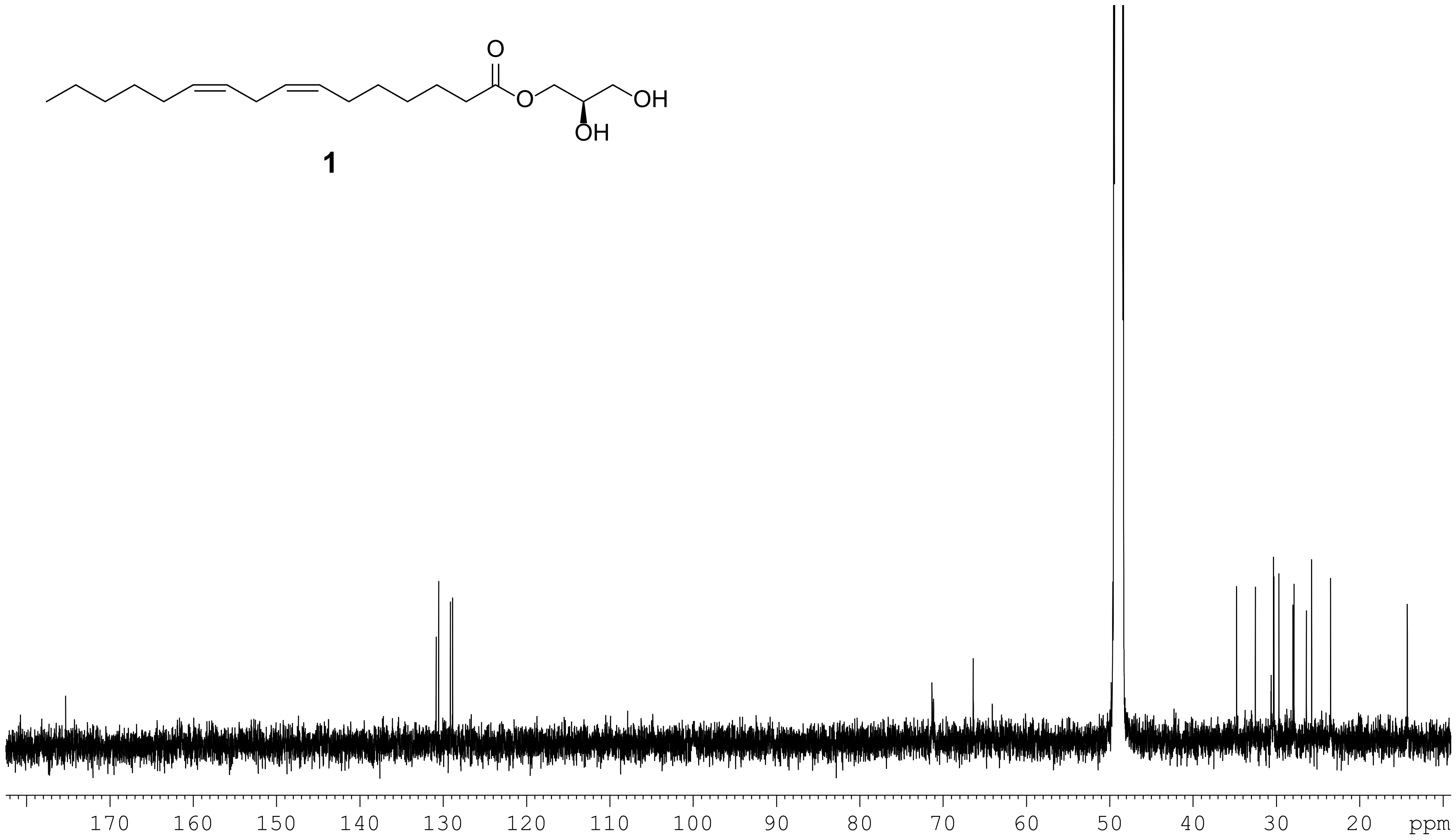

^{13}C NMR spectrum of nostochopcerol (**1**) (125 MHz, CD_3OH)

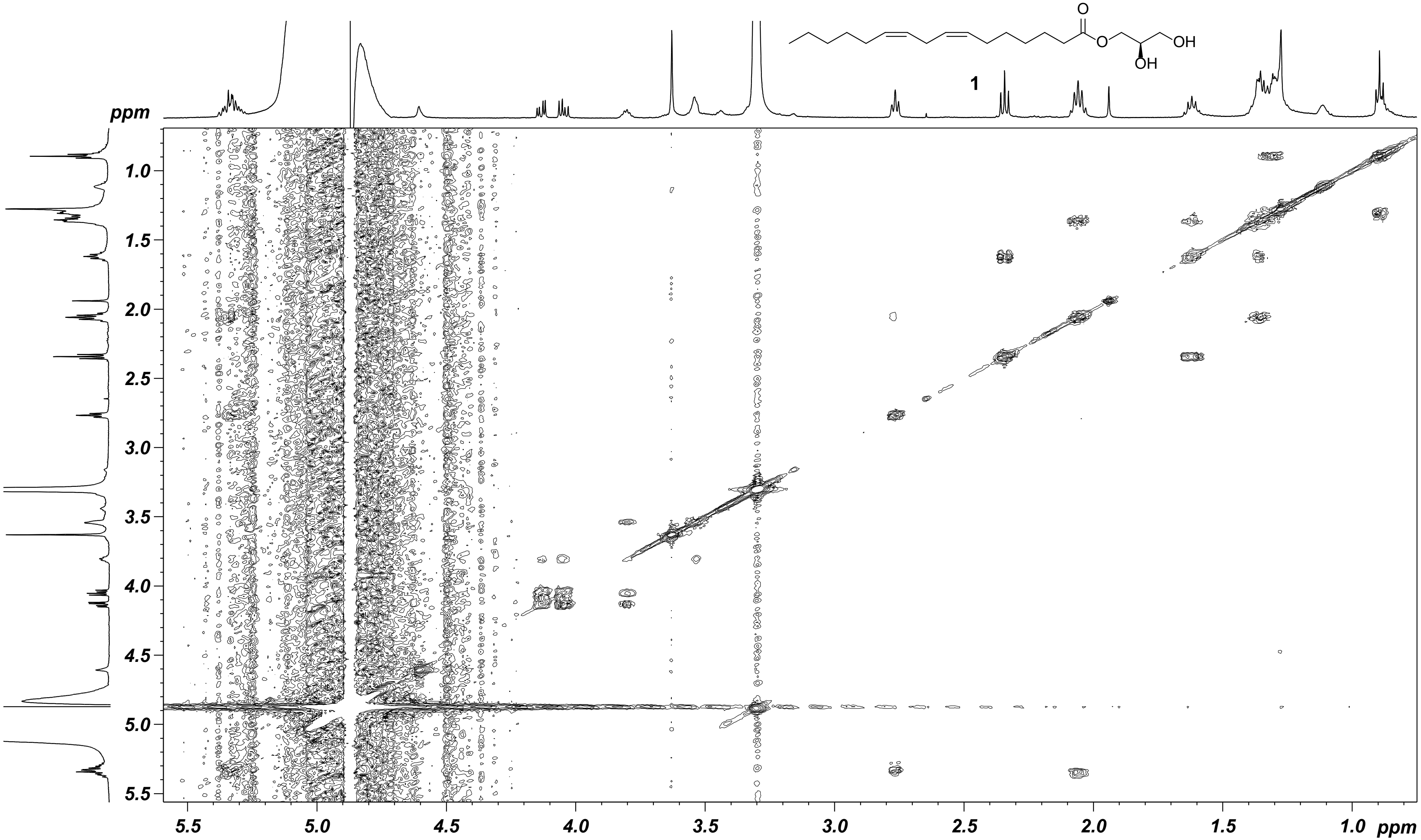

COSY spectrum of nostochopcerol (**1**) (500 MHz, CD_3OH)

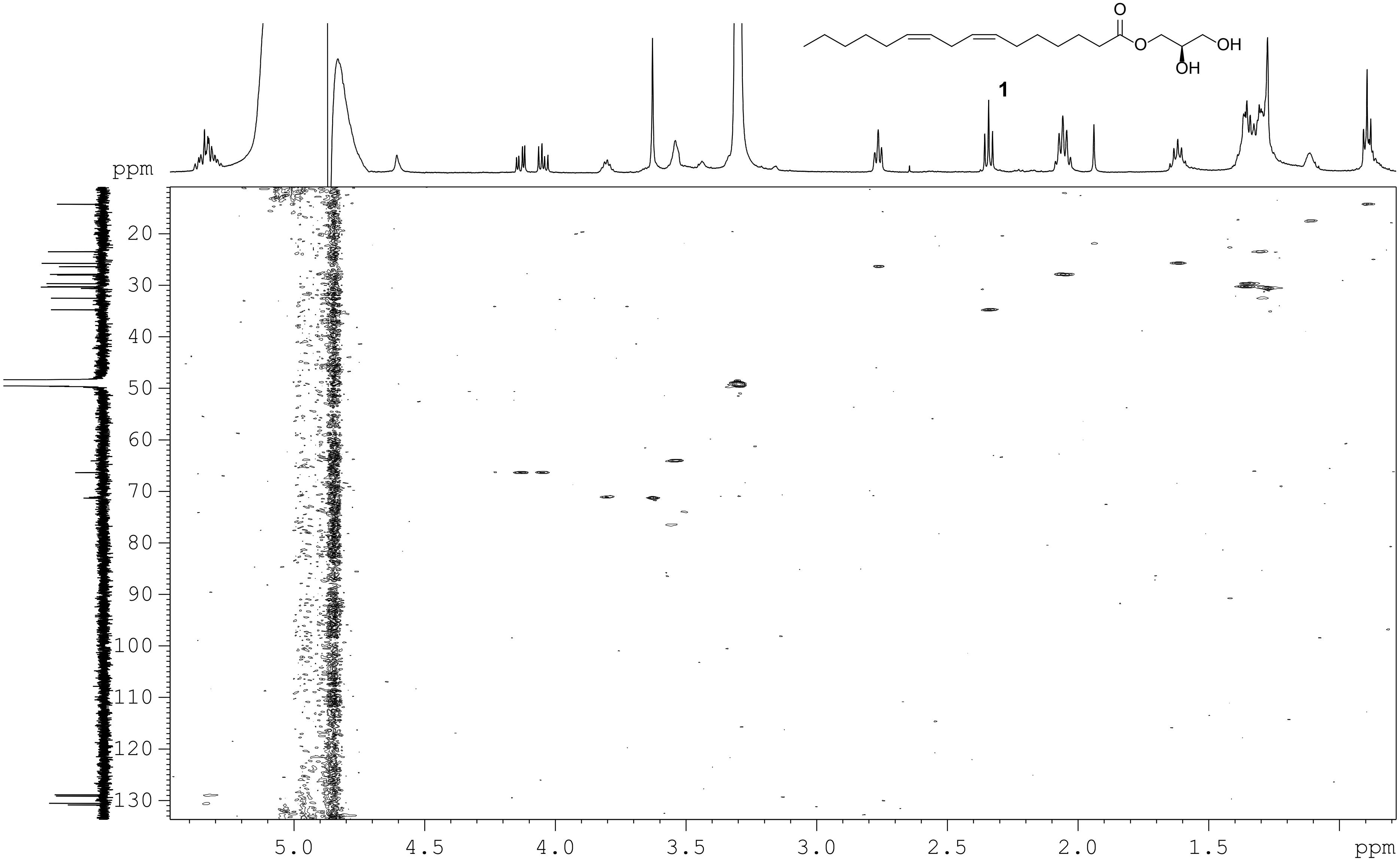

HSQC spectrum of nostochopcerol (**1**) (500 MHz, CD_3OH)

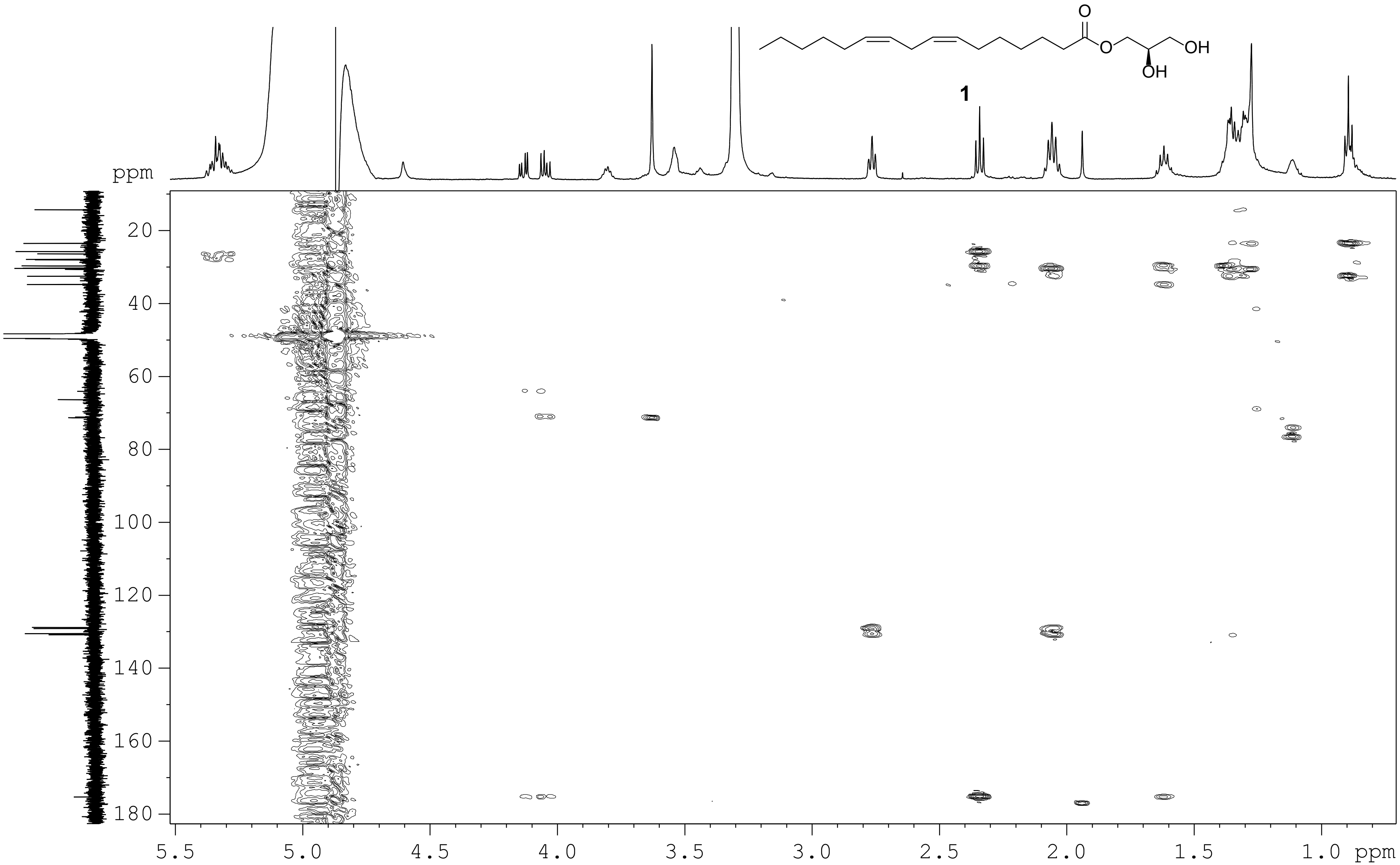

HMBC spectrum of nostochopcerol (**1**) (500 MHz, CD_3OH)

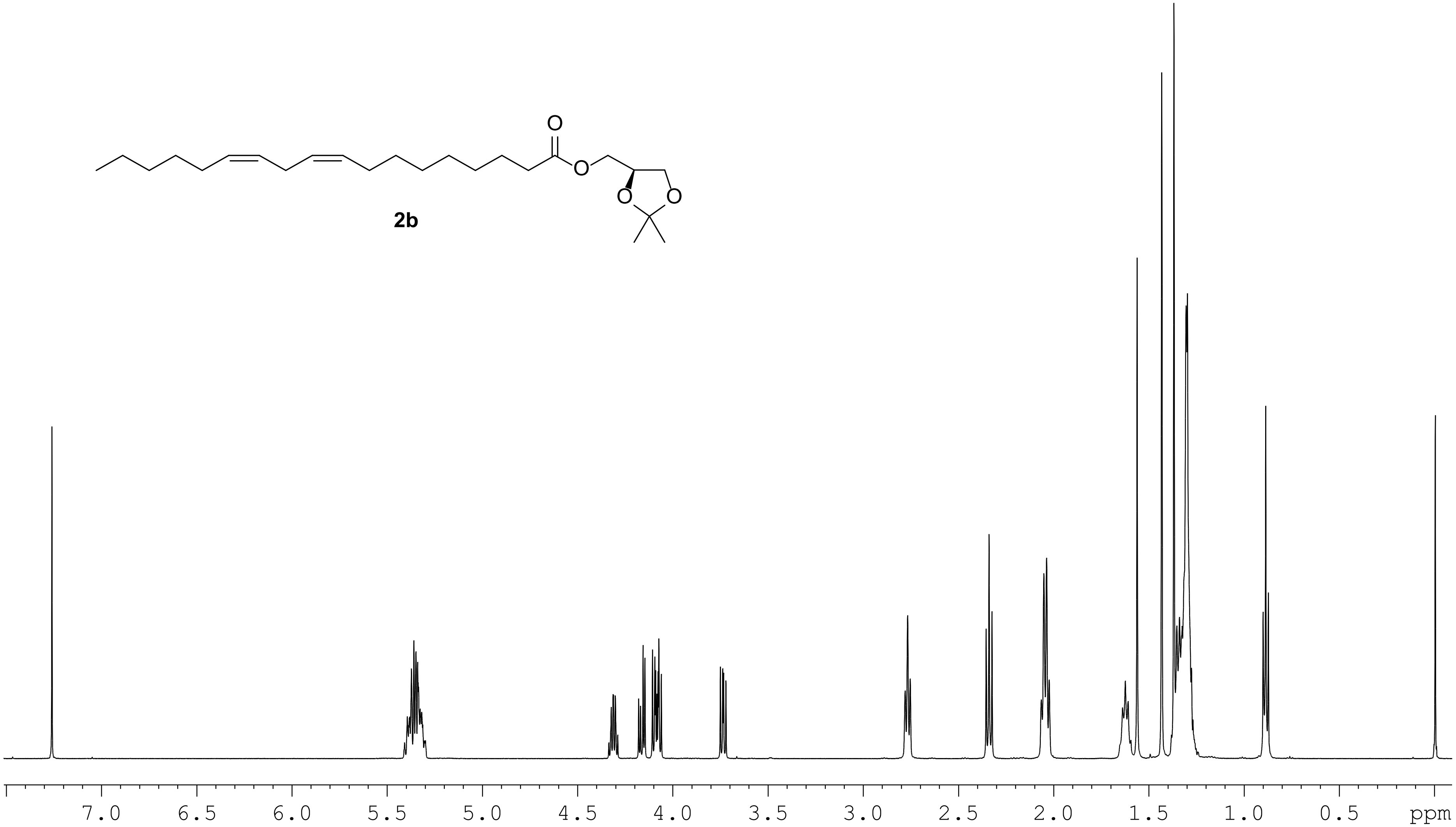

2b

^{1}H NMR spectrum of 3-linoleoyl-1,2-*O*-isopropylidene-*sn*-glycerol (**2b**) (500 MHz, $CDCl_3$)

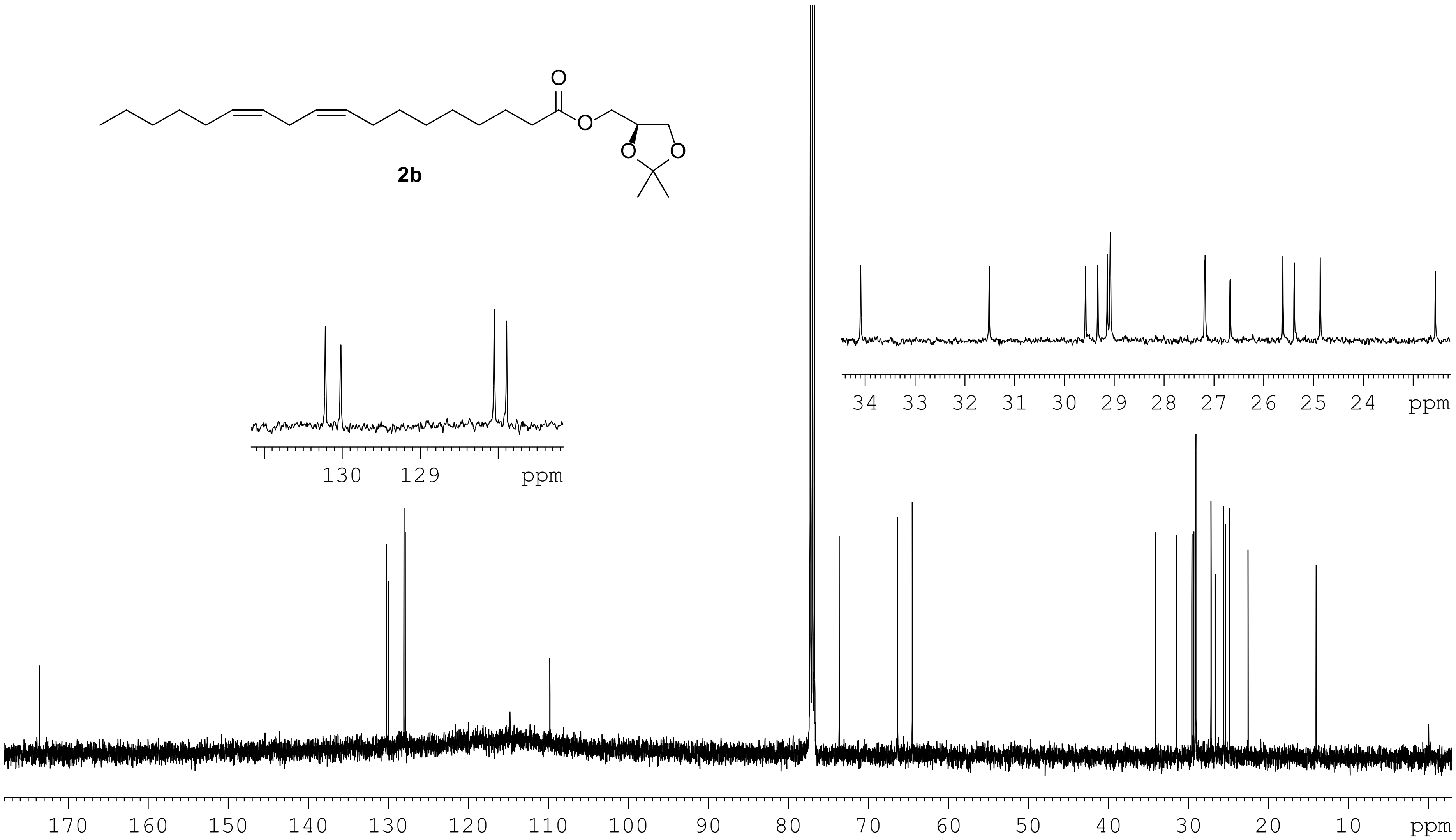

^{13}C NMR spectrum of 3-linoleoyl-1,2-*O*-isopropylidene-*sn*-glycerol (**2b**) (500 MHz, $CDCl_3$)

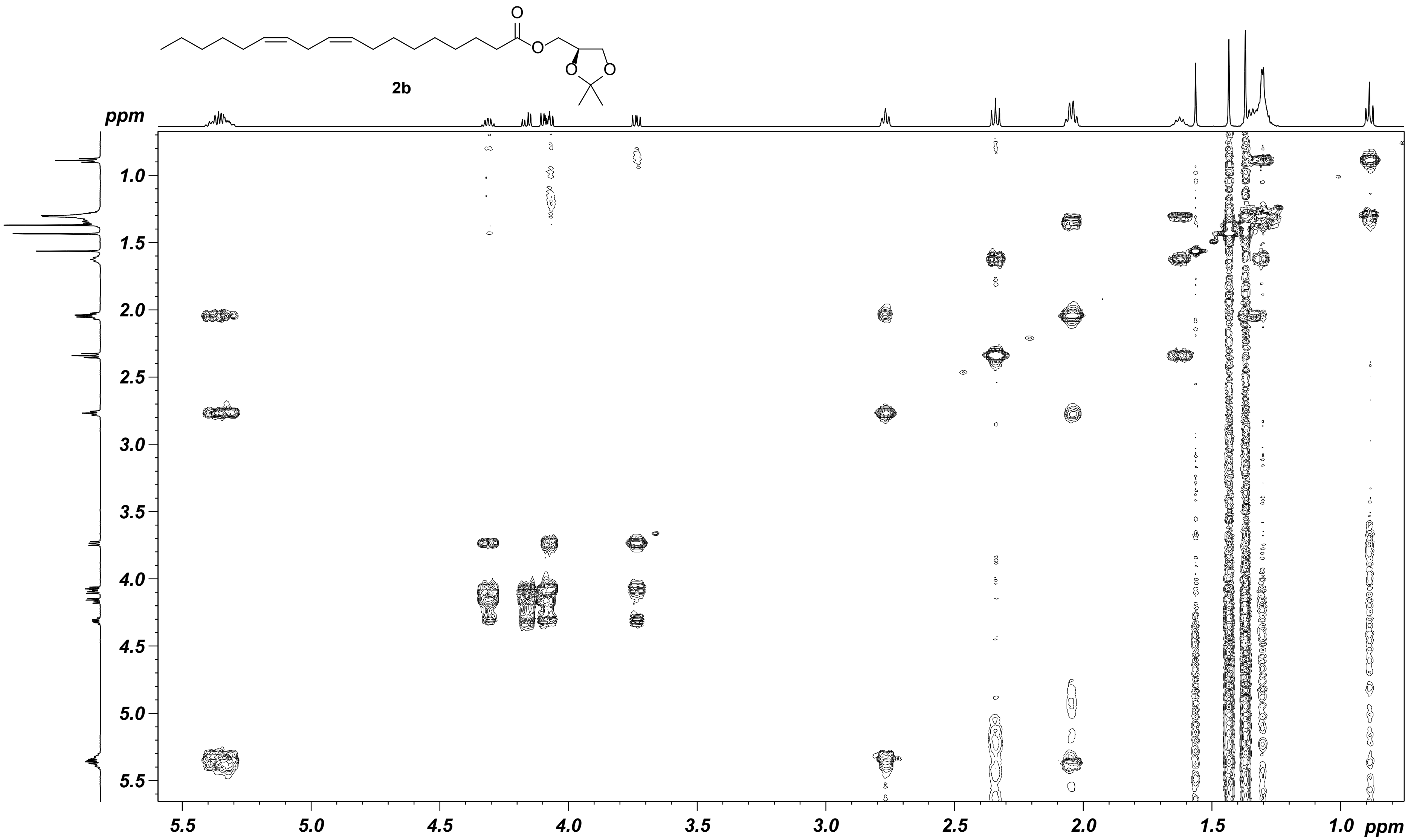

COSY45 spectrum of 3-linoleoyl-1,2-*O*-isopropylidene-*sn*-glycerol (**2b**) (500 MHz, $CDCl_3$)

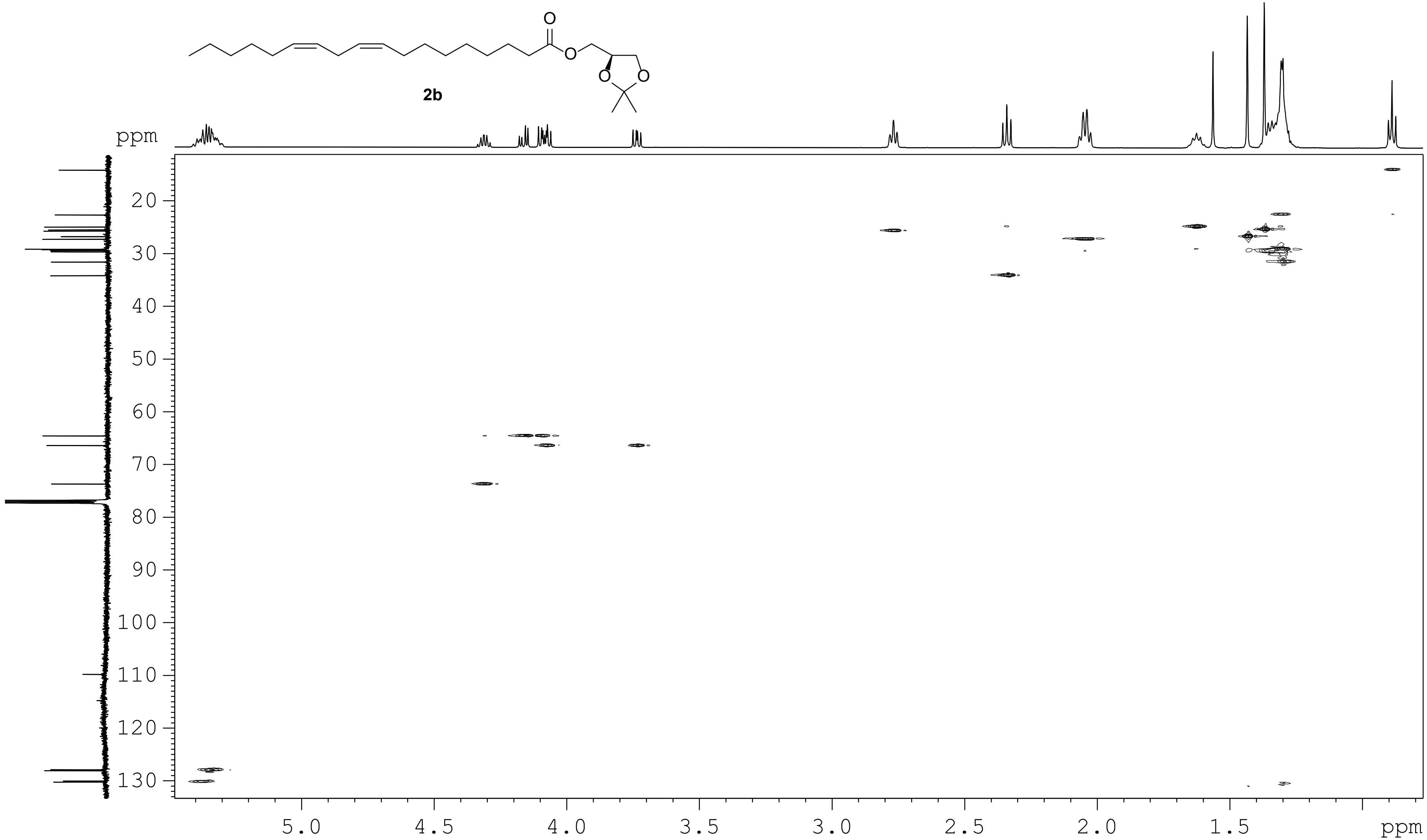

HSQC spectrum of 3-linoleoyl-1,2-*O*-isopropylidene-*sn*-glycerol (**2b**) (500 MHz, $CDCl_3$)

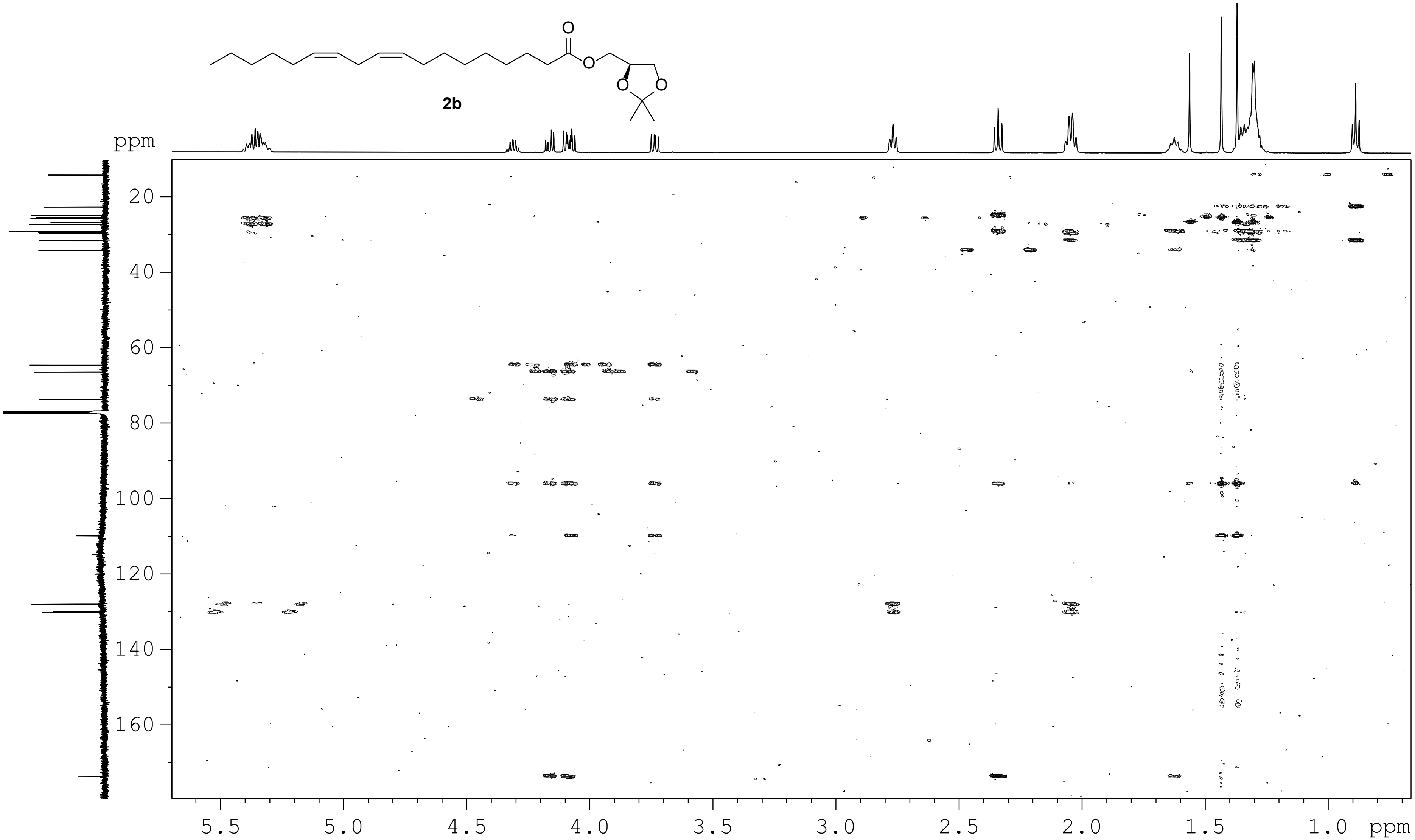

HMBC spectrum of 3-linoleoyl-1,2-*O*-isopropylidene-*sn*-glycerol (**2b**) (500 MHz, $CDCl_3$)

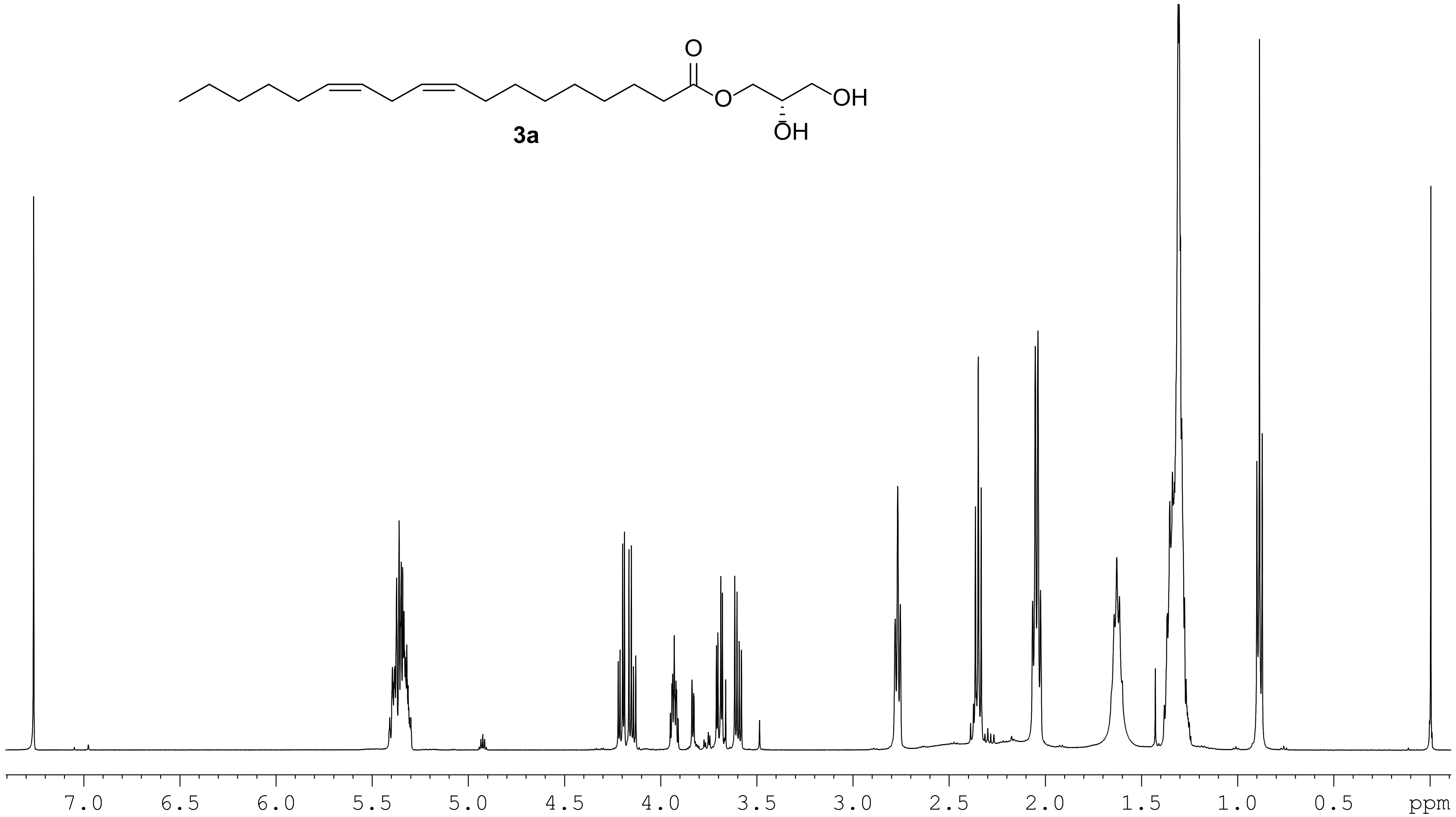

^{1}H NMR spectrum of 1-linoleoyl-*sn*-glycerol (**3a**) (500 MHz, $CDCl_3$)

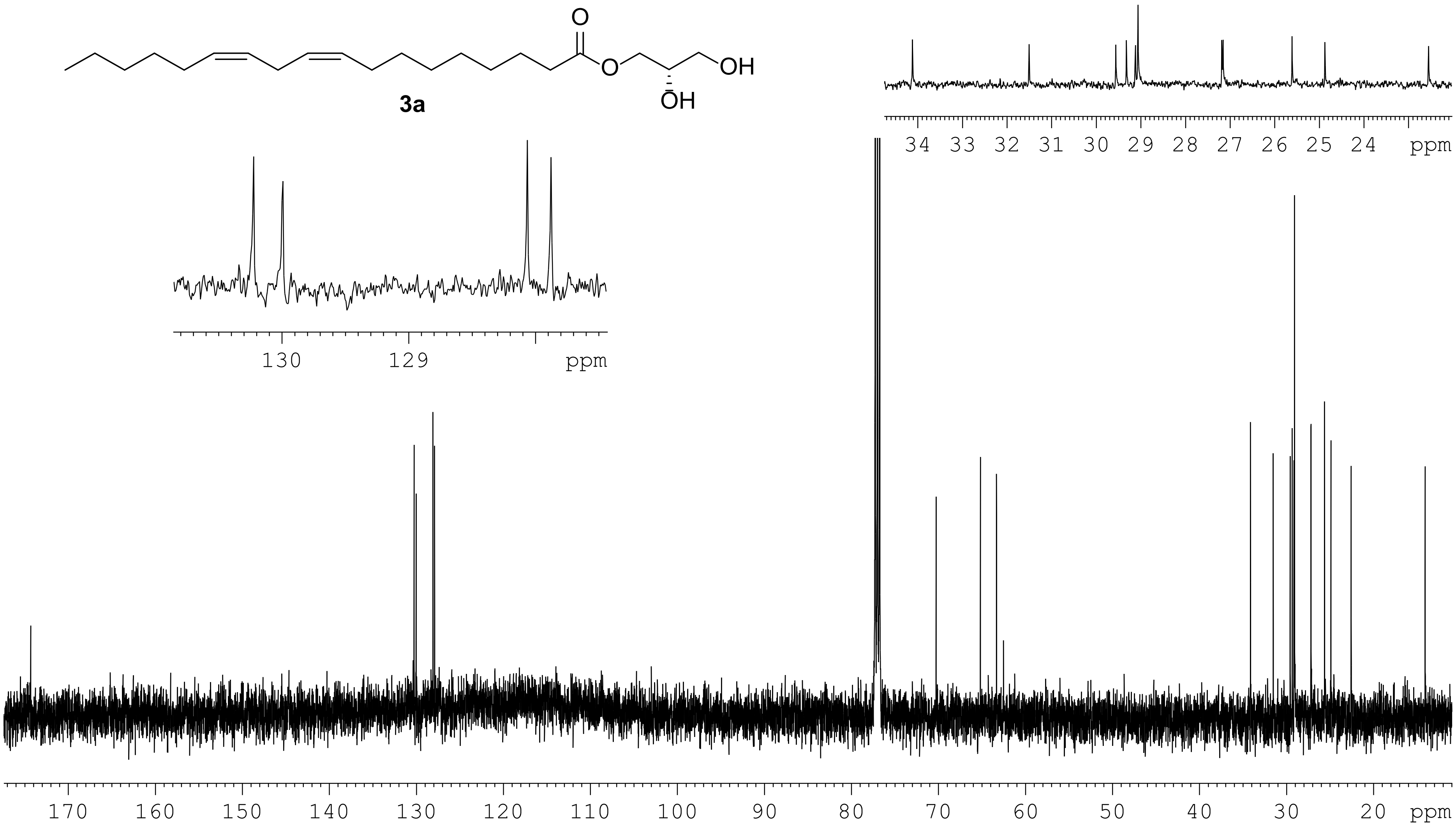

3a

^{13}C NMR spectrum of 1-linoleoyl-*sn*-glycerol (**3a**) (500 MHz, $CDCl_3$)

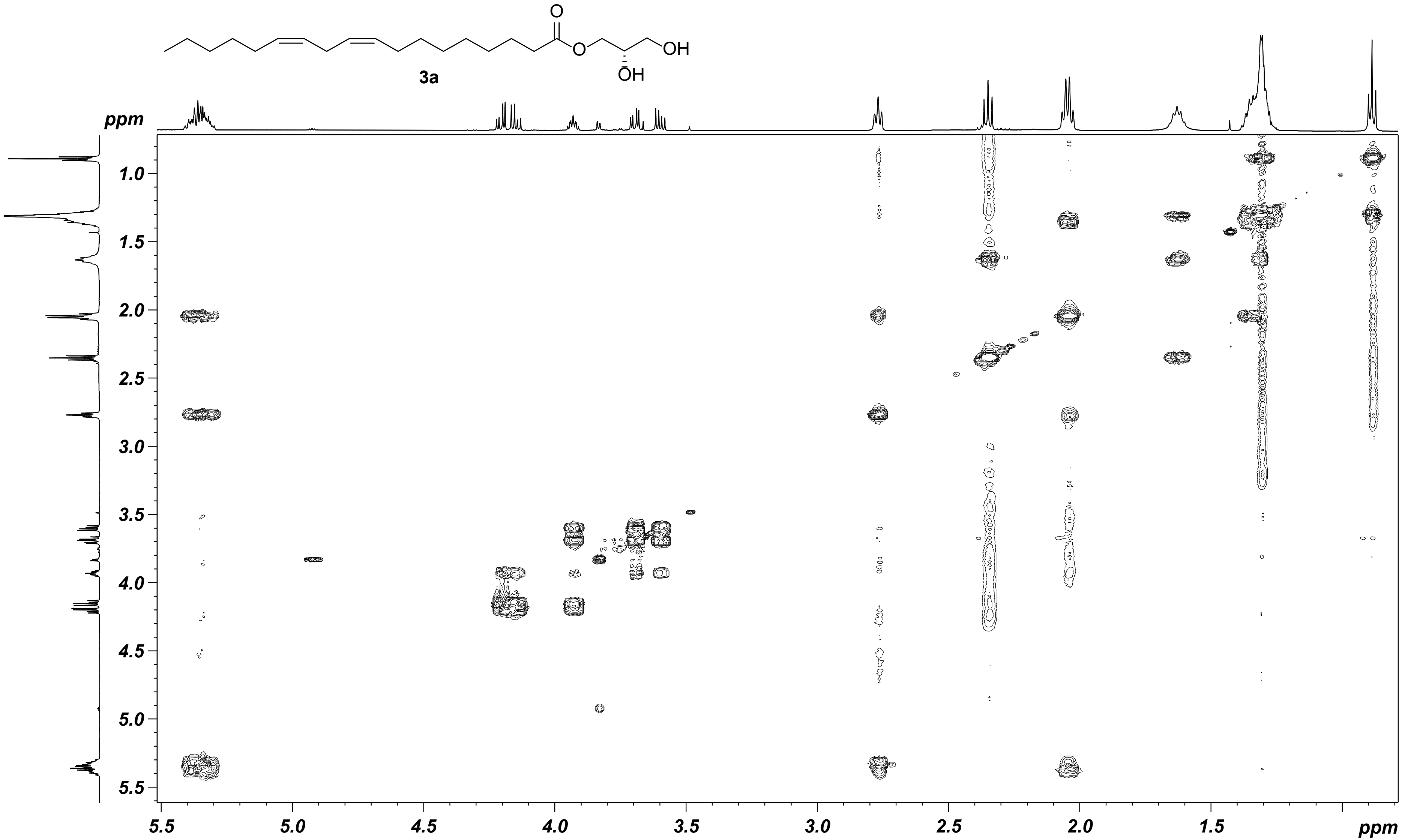

COSY45 spectrum of 1-linoleoyl-*sn*-glycerol (**3a**) (500 MHz, $CDCl_3$)

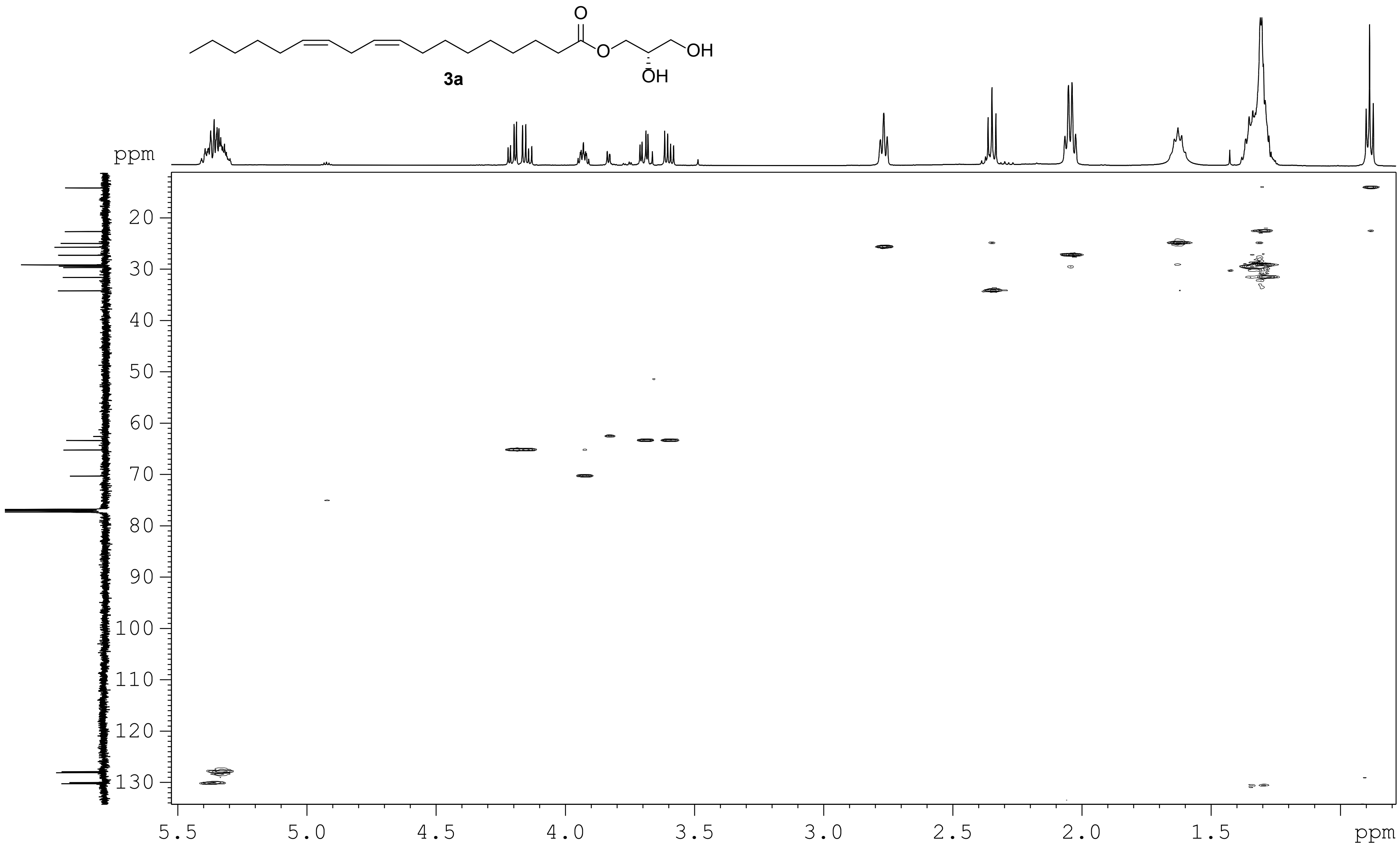

HSQC spectrum of 1-linoleoyl-*sn*-glycerol (**3a**) (500 MHz, $CDCl_3$)

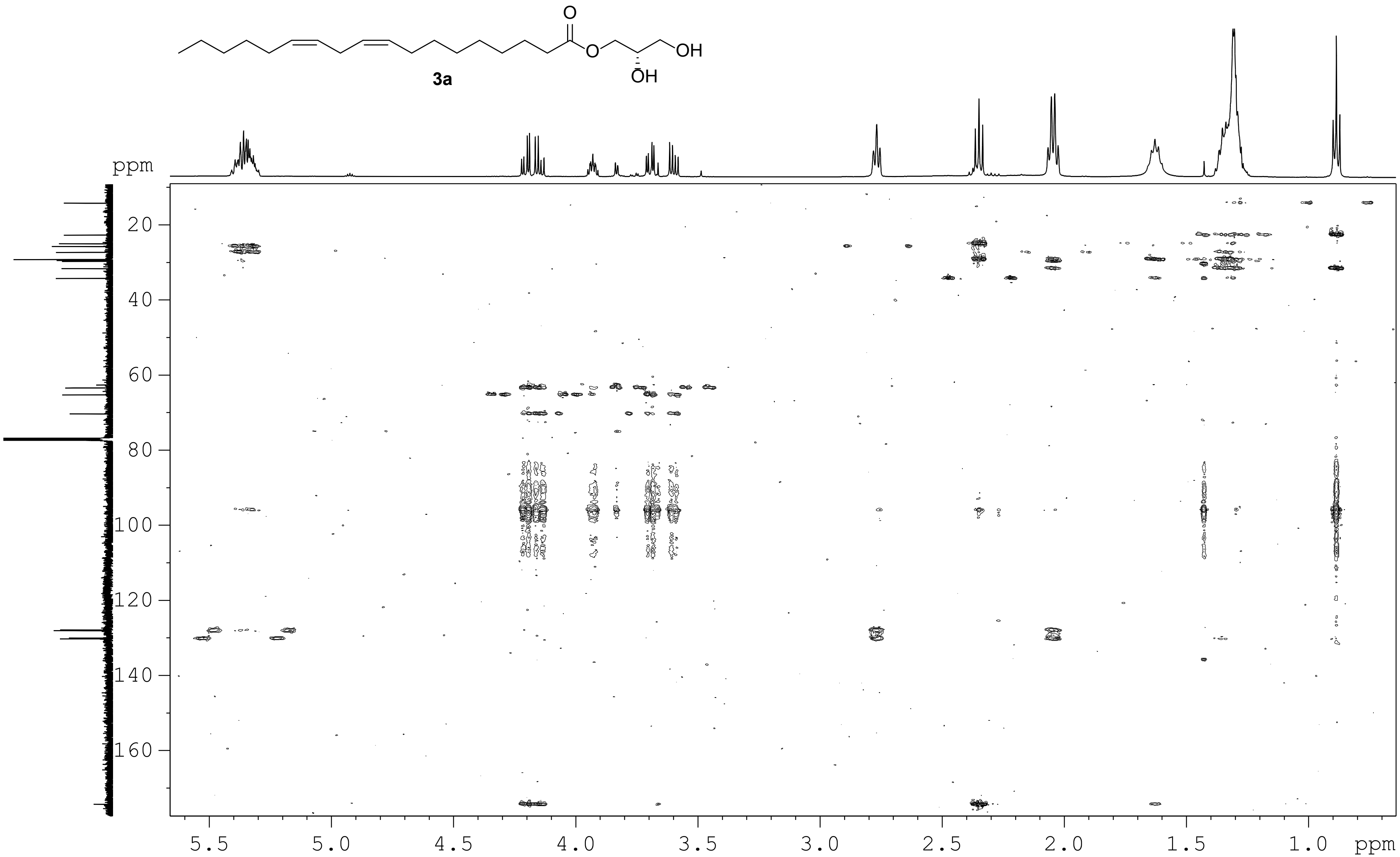

HMBC spectrum of 1-linoleoyl-*sn*-glycerol (**3a**) (500 MHz, $CDCl_3$)
